# *Entamoeba histolytica* and pathogenesis: A calcium connection

**DOI:** 10.1371/journal.ppat.1008214

**Published:** 2020-05-07

**Authors:** Mrigya Babuta, Sudha Bhattacharya, Alok Bhattacharya

**Affiliations:** 1 School of Life Sciences, Jawaharlal Nehru University, New Delhi, India; 2 School of Environmental Sciences, Jawaharlal Nehru University, New Delhi, India; 3 Department of Biology, Ashoka University, Sonepat, Haryana, India; Institut Pasteur, FRANCE

## Abstract

Calcium signaling plays a key role in many essential processes in almost all eukaryotic systems. It is believed that it may also be an important signaling system of the protist parasite *Entamoeba histolytica*. Motility, adhesion, cytolysis, and phagocytosis/trogocytosis are important steps in invasion and pathogenesis of *E*. *histolytica*, and Ca^2+^ signaling is thought to be associated with these processes leading to tissue invasion. There are a large number of Ca^2+^-binding proteins (CaBPs) in *E*. *histolytica*, and a number of these proteins appear to be associated with different steps in pathogenesis. The genome encodes 27 EF-hand–containing CaBPs in addition to a number of other Ca^2+^-binding domain/motif-containing proteins, which suggest intricate calcium signaling network in this parasite. Unlike other eukaryotes, a typical calmodulin-like protein has not been seen in *E*. *histolytica*. Though none of the CaBPs display sequence similarity with a typical calmodulin, extensive structural similarity has been seen in spite of lack of significant functional overlap with that of typical calmodulins. One of the unique features observed in *E*. *histolytica* is the identification of CaBPs (EhCaBP1, EhCaBP3) that have the ability to directly bind actin and modulate actin dynamics. Direct interaction of CaBPs with actin has not been seen in any other system. Pseudopod formation and phagocytosis are some of the processes that require actin dynamics, and some of the amoebic CaBPs (EhC2Pk, EhCaBP1, EhCaBP3, EhCaBP5) participate in this process. None of these *E*. *histolytica* CaBPs have any homolog in organisms other than different species of Entamoeba, suggesting a novel Ca^2+^ signaling pathway that has evolved in this genus.

## Introduction

The protist parasite *Entamoeba histolytica* causes human amebiasis, a major public health problem in developing countries. Though great strides have been made in understanding the pathobiological mechanisms of the disease in the last few decades, details about the molecular pathways that are involved in tissue invasion and damage during both intestinal and extraintestinal diseases are not clear. Because only a fraction of infected individuals (about 10%) display invasive disease, an understanding of the signaling system that triggers invasion by the parasite is needed for the development of better therapeutic molecules. Clear linkage between the genotype of the parasite with invasive disease or with extraintestinal invasion has not been seen, though a number of virulence factors have been identified in recent years [1]. The host–parasite relationship in amebiasis is also modulated by host factors, which include host genes (such as leptin) and gut microflora [2]. Gut bacteria provide not only feeding material but also an anaerobic environment and pH conducive for the trophozoites to multiply and differentiate into cysts [3]. It is increasingly believed that the gut environment and parasite genotype, along with the host genotype, all interact to create the right environment for *E*. *histolytica* to invade [3,4]. However, we do not have any clear idea about the nature of these interactions and how these eventually influence the parasite’s ability to invade tissues.

## Ca^2+^ homeostatic mechanism in *E*. *histolytica*

**Fig 1:**
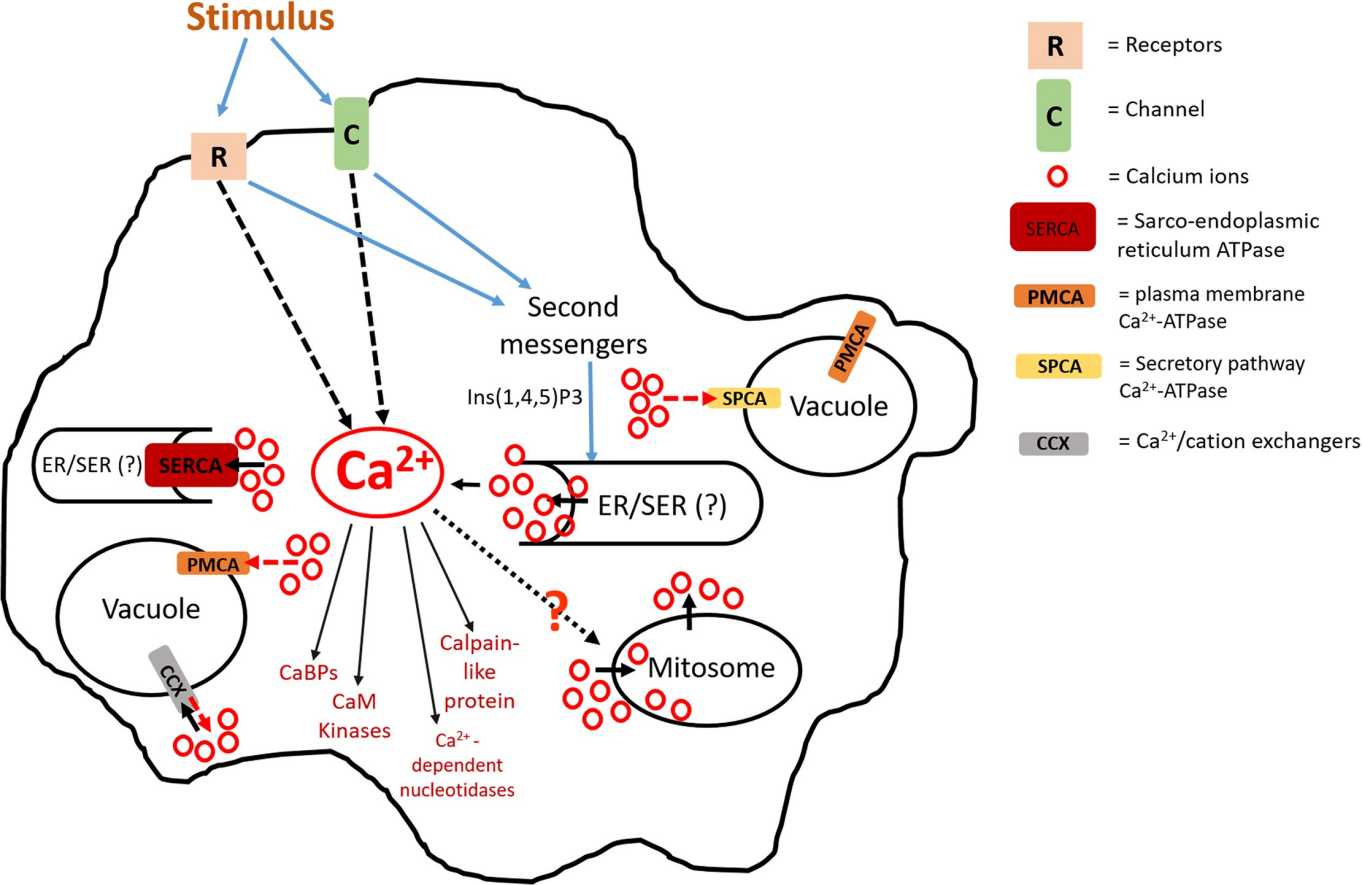
Model depicting the intracellular calcium dynamics in *E*. *histolytica*. Model summarizes the possible pathways of intracellular calcium dynamics after stimulus. The activation of receptor/channel leads to release of calcium from the ER-like structures into the cytosol. Second messengers such as Ins(1,4,5)P3 helps in the release of the internal Ca^2+^ pool in the cytosol. CaBPs, calpain-like protein, Ca^2+^-nucleotidase, CaM kinases, and other proteins modulates these Ca^2+^ signals into cellular response. Later on, Ca^2+^ is sequestered back into the internal sources by Ca^2+^-ATPase pumps such as SERCA and PMCA, which are located on the membrane of ER-like structures and in vacuoles, respectively. CCXs and SPCAs are other pumps localized on the vesicles of many organisms. Dotted arrows (in red) show the speculated Ca^2+^ ion flow. Because of the lack of evidence, it is difficult to speculate on the localization of different pumps on the vesicles, and the schematic diagram has been simplified for clarity. CaBP, Ca^2+^-binding protein; CaM kinases, Ca^2+^ modulated protein kinases; CCX, Ca^2+^/cation exchanger; ER, endoplasmic reticulum; Ins(1,4,5)P3, inositol 1,4,5-triphosphate; PMCA, plasma membrane Ca^2+^-ATPase; SER, smooth endoplasmic reticulum; SERCA, sarco/endoplasmic reticulum ATPase; SPCA, secretory pathway calcium ATPase.

Ca^2+^ is one of the versatile, ubiquitous second messengers that mediate pathways by altering the shape, charge, and electrostatic interaction of downstream effector molecules [5]. In order to mediate response in the presence of a stimulus, cells have developed a “signaling toolkit” to sequester or compartmentalize Ca^2+^ and release it as and when needed [6]. This toolkit comprises Ca^2+^-mobilizing signals that regulate the level of Ca^2+^ in different cellular compartments by activating various ion channels and transporting systems. Once Ca^2+^ is released, a repertoire of CaBPs, Ca^2+^ buffers, and Ca^2+^-regulated enzymes subtly translate these Ca^2+^ signals into a cellular response. After initiation of a response by activating the appropriate pathway, Ca^2+^ is rapidly removed from the cytoplasm by various pumps and exchangers [6]. It is not clear whether *E*. *histolytica* encodes most of the molecules needed for release and sequestering of Ca^2+^ in response to a signal. Only a handful of molecules have been reported. A figure summarizing our current understanding and the molecules involved is shown in Fig 1. There are 5 genes encoding putative Ca^2+^-ATPases, out of which 3 belong to plasma membrane Ca^2+^-ATPase (PMCA) and 2 to sarcoendoplasmic reticulum ATPase (SERCA), and these are present in vacuoles and in the cytoplasmic network, respectively [7,8]. More recently, 2 Ca^2+^-ATPases from *E*. *histolytica* (Eh), namely EhSPCA (secretory pathway calcium ATPase) and EhCCX (Ca^2+^/cation exchanger), have been identified. These are present on the membrane of some cytoplasmic vesicles [9,10]. Interestingly, overexpression of EhCCX enhanced the virulence and reduced the cell death of trophozoites [9].

*E*. *histolytica* has ionophore-releasable Ca^2+^, comprising around 70% of the total Ca^2+^ pool that can be divided into 2 parts. One is stimulated by the second messenger inositol 1,4,5-triphosphate (Ins(1,4,5)P3) releasing internal Ca^2+^ from endoplasmic reticulum-like structures [11]. The second one is sensitive to Ins(1,3,4,5)P4 [12]. Though it appears that both these second messengers act on 2 different Ca^2+^ stores, it is not clear whether there is a link between them in this organism. *E*. *histolytica* also encodes a calpain-like protein and many nucleotidases that require Ca^2+^, such as Ca^2+^-dependent ATPase/ADPase, Ca^2+^-dependent thiamine pyrophosphatase, and acid phosphatase. The calpain-like protein is thought to be associated with apoptosis of the parasite because its level is increased during programmed cell death of trophozoites. It was also found in the cytoplasm and near the nucleus [13,14], whereas some of the nucleotidase enzymes are present in the inner membrane of cytoplasmic vacuoles that may or may not be phagolysosomes [15–17]. It is also not clear whether these enzymes participate in calcium homeostasis in this organism. Genomic analysis identified a repertoire of 27 multi-EF-hand–containing CaBPs in *E*. *histolytica* [18]. Some of these proteins are suspected to be Ca^2+^ buffers, thereby participating in the regulation of Ca^2+^ concentration in different cellular compartments.

## Role of Ca^2+^ in the pathogenesis of *E*. *histolytica*

The initial step during the process of invasion is adherence to the target cells after the contact has been made. A number of molecules that are involved in this process have been identified. The most prominent among them is galactose-and *N*-acetyl-d-galactosamine (Gal/GalNAc) lectin, a 260-kDa heterodimeric cell-surface protein consisting of a 170-kDa heavy chain (hgl) bound to a 35/31-kDa light chain (Igl) through disulfide linkage [19]. The light subunit is thought to attach to the membranes through glycophosphoinositol anchors. The 260-kDa lectin is in complex with a 150-kDa intermediate subunit. The heavy chain has the carbohydrate recognition domain (CRD) displayed at the cell surface [20]. CRD recognizes target cells and ligand molecules through this domain. Overexpression of a mutant heavy chain subunit lacking essentially extracellular N-terminal domains (that is, mostly CRDs) conferred a dominant negative phenotype displaying reduced adherence and virulence in animal models [21]. Light subunits are also involved in adherence and virulence. Expression of mutated forms of lgl (part of C-terminal deletion) showed a dominant negative phenotype. The heterodimeric complex of mutant lgl with hgl is formed, but this complex is functionally inactive [22]. The glycosyl-phosphatidyl-inositol (GPI) anchor of lgl may be important for the formation of a complex with hgl because an expression of C-terminal (GPI anchor region)-deleted molecules did not lead to a 260-kDa complex. In addition to Gal/GalNAc lectin, a number of other cell-surface molecules that are involved in adherence have been identified. Among these, Lysine and glutamic acid-rich protein 1 (KERP1) and cysteine protease adhesin (CPADH112) have been described in more detail [23].

Ca^2+^ also participates in the binding of ligands by Gal/GalNAc lectin [20]. CRD has a Ca^2+^ binding site, and in one study, it was shown that Ca^2+^ binding is required for interaction with the ligand [24]. In a more recent study, a Ca^2+^ binding site was identified, and a mutant that lost the ability to bind Ca^2+^ was generated. Though carbohydrate-binding function was retained by the mutant protein, the ability to agglutinate red blood cells (RBCs) was lost, suggesting that some properties of Gal/GalNAc lectin are modulated by Ca^2+^ ions [25]. The Ca^2+^-binding chaperone protein calreticulin (CRT) is an *E*. *histolytica* cell-surface protein that binds complement component 1q (C1q) [26]. It participates in the phagocytosis of apoptotic immune cells, but not adherence or killing of normal cells such as Chinese hamster ovary (CHO) cells [26]. Moreover, the 2.15-Å X-ray structure of EhCRT showed a closed conformation of CRT with the dual carbohydrate and/or protein substrate-binding properties of lectin and that of chaperonin [27]. The pathway does not appear to be through Gal/GalNAc lectin and provides an alternate cell-surface–interacting system regulated by Ca^2+^.

## Cytolysis of target cells

It has been shown very clearly that adherence of *E*. *histolytica* to target cells is required for subsequent cell lysis and tissue invasion [28–30]. Death of the target cells can be directly mediated through hydrolytic and toxin molecules of *E*. *histolytica* or through stimulation of apoptotic pathway initiated after contact with the parasitic cells [31]. Amebic cells encode and express a large number of different genes that have proteolytic activity [32]. Among these, cysteine proteinase 5 (Ehcp5) has gained attention because it is located on the cell surface and because of the absence of a functionally active homolog in the nonpathogenic species *E*. *dispar* [33]. Porin-like proteins of *E*. *histolytica*, amebapores, were also implicated in cytolysis carried out by amebic cells [34–36]. One of the consequences of the interaction of *E*. *histolytica* with target cells is a dramatic rise of Ca^2+^ levels in the latter after contact. Blocking target cell Ca^2+^ channels inhibited cell death. This is thought to be initiated by Gal/GalNAc lectin because the purified protein itself enhances Ca^2+^ levels in target cells [29,37]. However, the mechanism of target cell Ca^2+^ release on contact with *E*. *histolytica* is not clear.

A number of studies suggest that Ca^2+^ signaling is also involved in the ability of *E*. *histolytica* to initiate target cell killing. Blocking the rise of intracellular Ca^2+^ in the parasite prevents the initiation of the process of cytolysis [28,29,38,39]. Direct involvement of Ca^2+^ in amebic virulence was seen when a Ca^2+^-binding transcription factor upstream regulatory element 3 binding protein (URE3BP) was found to regulate gene expression of virulence-associated genes. UREBP binds the promoter element of Gal/GalNAc lectin gene hgl5 [40,41]. It has 2 Ca^2+^-binding EF-hand motifs and negatively regulates transcription in presence of Ca^2+^; that is, it binds the promoter DNA motif only in the absence of Ca^2+^ [40,42]. A Ca^2+^-binding–defective mutant displayed a dominant positive phenotype, and cells expressing the mutant protein were more virulent [43]. URE3BP is likely to have a much wider role because a large fraction of amebic genes contain the URE3 motif recognized by URE3BP, thereby controlling expression of a number of genes [43]. URE3BP also shows an unusual localization at the plasma membrane of trophozoites apart from that in the nucleus. Membrane association is regulated by a 22-kDa Ca^2+^-dependent binding partner known as EhC2A [44]. Apart from these proteins, the ameba also displays a Ca^2+^-dependent phospholipase activity that may have a role in virulence [45].

## Phagocytosis and trogocytosis

Phagocytosis is intimately associated with the biology of *E*. *histolytica*. It displays a high rate of pinocytosis and phagocytosis that results in plasma membrane renewal every 30 min [46]. It phagocytoses a number of different cells that include RBCs, mammalian live and apoptotic cells, and bacterial cells. A number of reports have pointed out that phagocytosis plays a critical role in amebic virulence. Most of the evidence is based on the observed direct positive relationship of virulence potential with the phagocytic ability of an isolate. Generally, low phagocytic potential is correlated with less virulence. Moreover, a mutant defective in phagocytosis was found to be avirulent [47,48]. When this mutant was analyzed, it was observed that the level of EhCaBP1 was reduced several-fold in this mutant, suggesting that EhCaBP1 may be involved in phagocytosis [49]. Moreover, the essential role of Ca^2+^ in phagocytosis was also seen when chelation of Ca^2+^ in the cytoplasm led to inhibition of the process [50,51]. In the last few years, results from a number of studies have helped to outline a tentative molecular pathway of phagocytosis in *E*. *histolytica* [57]. It is clear from all these studies that Ca^2+^ plays a critical role from the initiation stage to the formation of phagosomes.

Phagocytosis is initiated by the recruitment of a C2-domain–containing protein kinase (EhC2PK) at the particle attachment site [52]. This recruitment requires Ca^2+^ and C2 domains and takes place when C2 binds membranes in the presence of Ca^2+^ [52,53]. We believe that the recruitment and enrichment of EhC2PK is the trigger for further assembly of the phagocytosis complex that starts with cups proceeding towards phagosomes. The formation of the phagocytosis complex requires multiple EhCaBPs, namely EhCaBP1, EhCaBP3, and EhCaBP5. EhC2PK recruits EhCaBP1 at the phagocytic stage, and Ca^2+^ is not required at this step. Once EhCaBP1 is at the phagocytic initiation site, it binds and recruits the atypical protein kinase EhAK1 in presence of Ca^2+^ [54,55]. EhAK1 is responsible for recruiting actin-related protein (Arp 2/3) complex proteins through the subunit EhARPC1 [56]. Arp2/3 complex proteins in turn bind calmodulin-like CaBP EhCaBP3, and this step requires the presence of Ca^2+^ [57]. A typical calmodulin is thought to be absent in *E*. *histolytica* because no conserved gene has been seen in this system. EhCaBP3 is thought to be the closest homolog because it displays the highest degree of sequence similarity (about 49%) with calmodulins [58]. Both EhCaBP3 and EhCaBP5 bind atypical myosin 1B in the presence of Ca^2+^ [58,59]. Myosin 1B has been shown to be an important component of phagocytic machinery [46]. Imaging experiments have clearly shown that the myosin 1B–EhCaBP3 complex participates in the pseudopod fusion and subsequent separation from the membrane [58]. The role of EhCaBP5 in the context of its interaction with myosin 1B is not clear, though the results do indicate involvement in pseudopod fusion [59]. It is tempting to speculate that EhCaBP3 and EhCaBP5 regulate myosin 1B function and probably have different roles during phagocytosis [58,59]. Overexpression of Ca^2+^-binding–defective mutants of all these proteins helped to delineate the participation of Ca^2+^ in different steps. Generally, these mutants display a dominant negative phenotype with respect to phagocytosis. Interestingly, overexpression of Ca^2+^-binding–defective EhCaBP1 did not interfere with the formation of phagocytic cups or the process of recruitment, but the process of transition from cups to phagosomes was blocked, thereby indicating that EhCaBP1 recruitment is independent of Ca^2+^ [50]. Therefore, it appears that Ca^2+^ has both direct and indirect roles in the phagocytosis of *E*. *histolytica*.

Trogocytosis has recently been shown to be a novel mechanism of target cell killing and virulence of *E*. *histolytica* [60,61]. The trophozoites tend to ingest fragments of live human target cells that lead to target cell death. This process has been termed amebic trogocytosis [61]. The process is likely to be initiated through the AGC family kinase 1 gene that is present only during the trogocytic event, but not during phagocytosis [62]. Subsequently, an EhC2PK-mediated pathway, similar to that observed for phagocytosis, is involved in the process [61]. Therefore, Ca^2+^ also plays an important role in trogocytosis.

## Other CaBPs

**Fig 2:**
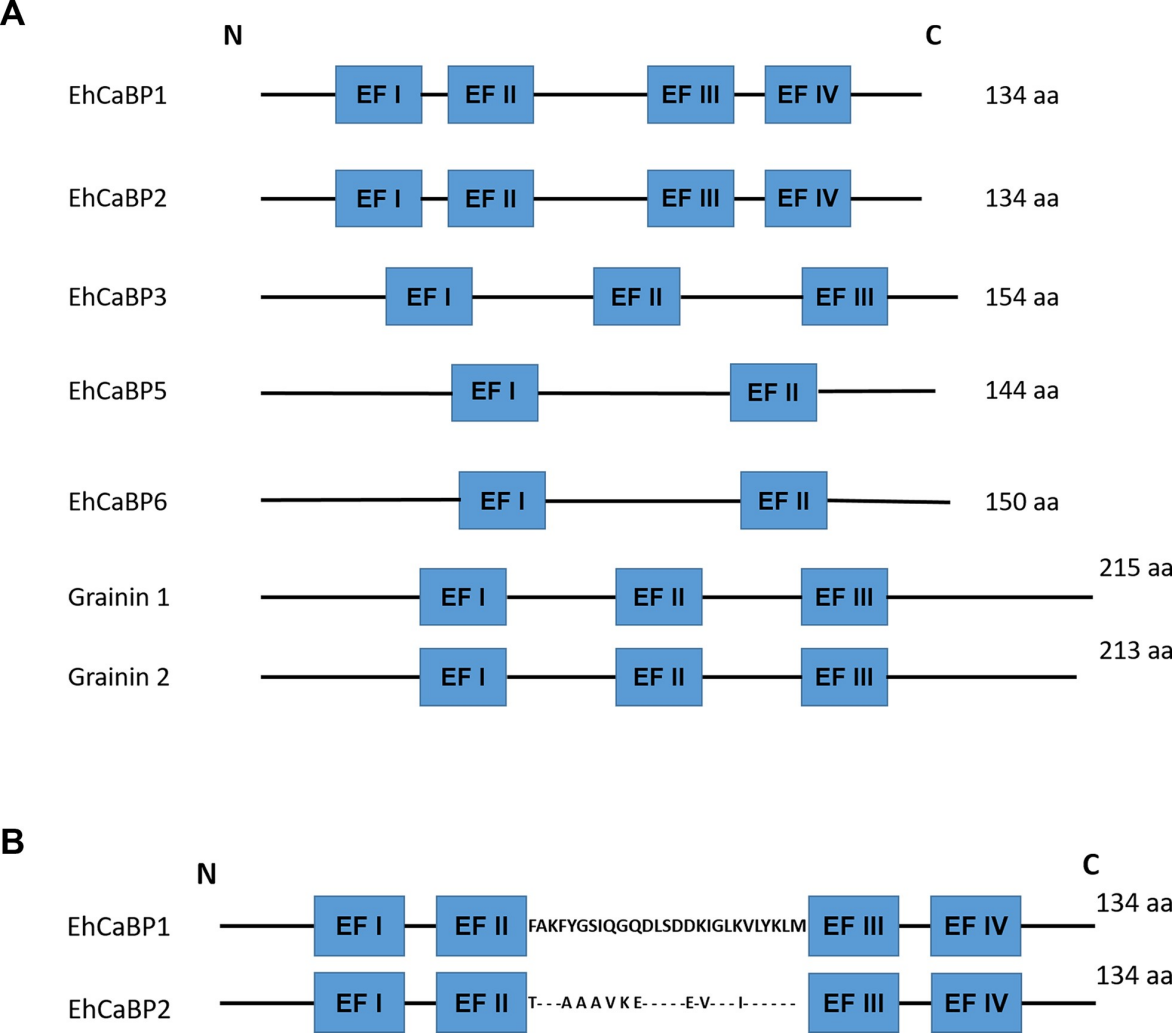
Domain organization of CaBPs. (A) Schematic representation of the domain organization of different CaBPs. (B) The linker regions of EhCaBP1 and EhCaBP2 between EF II and EF III are shown. Though EF sequences are highly conserved, the linker region sequences have extensive variation. aa, amino acid; CaBP, Ca^2+^-binding protein; EhCaBP, *E*. *histolytica* Ca^2+^-binding protein.

A number of as yet functionally uncharacterized CaBPs have been described in *E*. *histolytica*. The most prominent among these are 2 novel granule proteins grainin 1 and 2, which not only show a considerable structural similarity to EF-hand-motif–containing CaBPs but also bind Ca^2+^ [63–65]. These proteins are thought to be involved in vesicular maturation and exocytosis. However, there is no evidence in support of these activities. Recent studies have suggested the involvement of grainin 2 in amebic virulence because it was found to be present differentially in virulent organisms [63]. EhCaBP2 displays 79% sequence identity with EhCaBP1 and also has 4 Ca^2+^-binding EF-hand domains [66] (Fig 2). The central linker region between EF-hand domains 2 and 3 is most varied between EhCaBP1 and EhCaBP2 (Fig 2B). This region is thought to be involved in binding target molecules [66], suggesting that these 2 CaBPs are functionally different [67]. Unlike EhCaBP1, EhCaBP2 is involved in neither phagocytosis nor pseudopod formation. Moreover, these 2 proteins activate different sets of endogenous kinases and probably bind different sets of proteins in a Ca^2+^-dependent manner [66–68]. However, the functional role of EhCaBP2 is yet to be deciphered. A nuclear-localized CaBP, EhCaBP6, was also characterized [69,70]. It was found to be involved in cell division by modulating microtubule dynamics by increasing the rate of tubulin polymerization through binding to *E*. *histolytica* beta-tubulin.

## Conclusion and future directions

Calcium is known to be involved in many cellular processes in almost all eukaryotic systems. Therefore, it is not surprising that Ca^2+^ is also required for a number of processes, including pathogenesis in *E*. *histolytica*. The surprising part is the extensive participation of Ca^2+^ and CaBPs in a few systems such as phagocytosis not observed in any other eukaryotic systems (see Table 1). Moreover, direct involvement of CaBPs in regulating actin dynamics as shown in this organism is also quite unique. Evolution of this novel pathway regulating phagocytosis may be for adapting to a situation in which there is a very high rate of phagocytosis/endocytosis, leading to complete recycling of membrane every 30 min [71]. Analysis of live-cell imaging data does indicate that phagocytosis of a red blood cell is complete within 30 s after attachment. Ca^2+^ signaling is a rapid response, and mobilization of these EhCaBPs may not cause time delays after binding of a particle to the cell surface. Fast imaging of mobilization of different proteins during phagocytosis may help us to understand the nature of this rapid assembly process. The involvement of Ca^2+^ in many other amebic processes has not been investigated. Given the large number of CaBPs encoded by the *E*. *histolytica* genome, it will not be surprising to find Ca^2+^ signaling regulating a large number of pathways affecting the overall biology of *E*. *histolytica*.

**Table 1:** List of proteins that interact with calcium and play a role in amebic homeostasis and pathogenesis.

Name of Protein	Function/Role	Reference
EhPMCA	It is present in vacuoles and in cytoplasmic network; however, function is unknown.	[7]
EhSERCA	It is present in vacuoles and in cytoplasmic network. Function is unknown.	[8]
EhSPCA	Putative Ca^2+^-ATPase that is localized in vacuoles stained with NBD C6-ceramide, a Golgi apparatus marker. Function is unknown.	[10]
EhCCX	CCX that plays a role in programmed cell death and in virulence.	[9]
Ca^2+^-dependent ATPase/ADPase	They are localized in the inner membrane of cytoplasmic vacuoles that may or may not be phagolysosomes. Function is unknown.	[15,17]
Calpain-like protein	Ca^2+^-dependent cysteine protease involved in programmed cell death.	[13,14]
Ca^2+^-dependent thiamine pyrophosphatase	They are localized in the inner membrane of cytoplasmic vacuoles that may or may not be phagolysosomes. Function is unknown.	[16]
Gal/GalNAc	It is involved in the process of invasion because it helps in adhering to the target cells.	[19–22,24,25]
EhCRT	Amebic CRT is involved in the phagocytosis of apoptotic immune cells.	[26,27]
UREBP	It regulates the transcription of amebic genes and inhibits transcription in the presence of Ca^2+^.	[40,43,44]
EhC2A	It helps in localization of UREBP to the membrane apart from the nucleus.	[44]
EhC2PK	C2PK that is involved in initiation of phagocytosis.	[52,53]
EhCaBP1	Calcium-binding protein 1 that directly regulates erythrophagocytosis and actin dynamics.	[49,50,72]
EhCaBP2	It is 79% identical to EhCaBP1 but neither involved in phagocytosis or pseudopod formation. Function is not known.	[66–68]
EhCaBP3	Calcium-binding protein 3 interacts with the Myosin IB and Arp2/3 complex and plays a role in erythrophagocytosis.	[57,58]
EhCaBP5	Calcium-binding protein 5 is likely to be a light chain of myosin IB that is involved in phagocytosis.	[59]
EhCaBP6	Calcium-binding protein 6, which is involved in cell division and modulates microtubule dynamics.	[69,70]
Grainin1 and 2	EF-hand-motif–containing calcium-binding proteins involved in amebic virulence. It is also speculated they are also involved in vesicle maturation and exocytosis.	[63,64]
EhCaBP7–27	Other calcium-binding proteins encoded in the *E*. *histolytica* genome. Function is not deciphered yet.	[18]

**Abbreviations:** CaBP, Ca^2+^-binding protein; CCX, Ca^2+^/cation exchanger; CRT, calreticulin; C2PK, C2-domain–containing protein kinase; Eh, *E*. *histolytica*; Gal/GalNAc, galactose- and *N*-acetyl-d-galactosamine; NBD 6-ceramide, (6-((N-(7-nitrobenz-2-oxa-1,3-diazolyl)amino)hexanoyl)sphingosine); PMCA, plasma membrane Ca^2+^-ATPase; SERCA, sarcoendoplasmic reticulum ATPase; SPCA, secretory pathway calcium ATPase; UREBP, upstream regulatory element binding protein.
